# Machine Learning-Driven Automated Culture Reveals Similar Performance of mTeSR+ and StemFlex in Human iP11N Pluripotent Stem Cells

**DOI:** 10.3390/cells15171533

**Published:** 2026-08-25

**Authors:** Aleksander A. Bogoniewski, Melissa F. Gonzalez, Rachel E. Reyes, Susanna F. Rivera, Cameron Taylor, Todd Schurr, Robert Damoiseaux, Gerald S. Lipshutz

**Affiliations:** 1 Department of Molecular and Medical Pharmacology, David Geffen School of Medicine, University of California, Los Angeles (UCLA), Los Angeles, CA 90095, USA; 2 Department of Surgery, David Geffen School of Medicine, University of California, Los Angeles (UCLA), Los Angeles, CA 90024, USAsusannarivera87@gmail.com (S.F.R.); 3 Department of Biology, California State University, Northridge, Los Angeles, CA 91330, USA; 4 Broad Stem Cell Research Center, University of California, Los Angeles (UCLA), Los Angeles, CA 90095, USA; 5 California NanoSystems Institute, University of California, Los Angeles (UCLA), Los Angeles, CA 90095, USA; 6 Department of Bioengineering, University of California, Los Angeles (UCLA), Los Angeles, CA 90095, USA; 7 Jonsson Comprehensive Cancer Center, University of California, Los Angeles (UCLA), Los Angeles, CA 90024, USA; 8 Intellectual and Developmental Disabilities Research Center, University of California, Los Angeles (UCLA), Los Angeles, CA 90095, USA; 9 Semel Institute for Neuroscience, University of California, Los Angeles (UCLA), Los Angeles, CA 90024, USA

**Keywords:** human induced pluripotent stem cells, automated cell culture, machine learning, stem cell culture media

## Abstract

Human induced pluripotent stem cells (hiPSCs) are powerful tools for disease modeling and therapeutic development, though their utility remains sensitive to operator-dependent variability and media formulation. Commercial stem cell media formulations, such as mTeSR+ and StemFlex, are designed to support robust pluripotent stem cell maintenance and improve reproducibility. While both are widely used, direct comparisons are limited. We evaluated mTeSR+ and StemFlex using the CellXpress.ai automated tissue culture platform in combination with IN Carta machine-learning-based image analysis to monitor and standardize feeding, passaging, and maintenance. Wild-type iP11N hiPSCs were cultured under standardized automated conditions with growth kinetics, immunocytochemical marker expression, and RT-qPCR assessment. Automated analysis revealed no significant differences in proliferation rates between the formulations; however, longitudinal image-based quantification identified statistically significant differences (<1%) in differentiated cell area fraction across the imaging period. Immunocytochemistry demonstrated comparable expression and localization of pluripotency-associated markers, and RT-qPCR analysis confirmed similar expression of stemness factors. These findings demonstrate that both media support comparable growth and maintenance of pluripotency under standardized automated conditions on iP11N wild-type stem cells in the short-term culture conditions analyzed. Additionally, these data highlight the value of automated imaging and machine-learning-based analysis in reducing operator-dependent variability and improving consistency in hiPSC culture, supporting its application in high-throughput stem cell research.

## 1. Introduction

Human induced pluripotent stem cells (hiPSCs) are an indispensable tool in biomedical research due to their ability to self-renew and differentiate into all somatic cell lineages [1]. These properties enable patient-specific disease modeling and support the development of emerging cell-based therapeutic applications across diverse biological systems [2,3]. Maintaining hiPSC identity in vitro requires carefully defined culture conditions that minimize cellular stress and spontaneous differentiation [4]. The composition of hiPSC culture media is a major determinant of cellular proliferation, survival, maintenance of pluripotency, lineage propensity, and long-term genomic and phenotypic stability [5]. Consequently, media selection remains a critical experimental variable that directly influences stem cell state maintenance and overall reproducibility across downstream applications.

Media formulation modulates the signaling and metabolic networks that sustain pluripotency. Defined components, including growth factors, small-molecule pathway inhibitors, and trace elements, can influence TGF-β and FGF signaling dynamics, redox homeostasis, and early lineage priming states [6,7]. Over extended periods of culture, these effects can accumulate to influence genomic stability, epigenetic landscape, and secretome composition, impacting both experimental reproducibility and downstream differentiation outcomes [8].

A further limitation in comparing commercially available hiPSC media formulations is the proprietary nature of their compositions. While the foundational components of earlier media such as mTeSR1 were described in the primary literature [9], the precise formulation of current commercial products, including mTeSR+ and StemFlex, is not fully disclosed by their respective manufacturers and is protected as proprietary trade secrets. As a result, direct mechanistic comparisons between media formulations are constrained, and observed functional differences cannot be readily attributed to specific molecular constituents. This opacity limits the interpretability of media comparison studies and underscores the need for empirical, head-to-head evaluation of commercially available formulations under standardized conditions.

Stem cell culture media can be categorized as xeno-containing, incorporating animal-derived components which include fetal bovine serum (FBS), or xeno-free, which rely exclusively on recombinant or human-sourced factors to reduce immunogenic risk [10]. Media components, particularly serum or biologically derived supplements, introduce exogenous variables, including extracellular vesicles, bioactive proteins, cytokines, and small-molecule contaminants, that can confound downstream analyses in hiPSC-derived vesicle profiles, cytokine secretion, and broader secreted and intracellular molecular readouts [11,12]. Xeno-free formulations, while designed to reduce variability associated with animal-derived products, may still exhibit lot-to-lot differences that impact hiPSC quality and the consistency of derived cell populations [13]. Additionally, transitioning cell culture from xeno-containing to xeno-free conditions can alter progenitor-level phenotypes. McGrath et al. demonstrated that long-term culture of hiPSC-derived mesenchymal progenitors under xeno-free, GMP-compatible conditions resulted in modest transcriptional and cytokine profile changes, while mesenchymal surface marker expression and cell viability remained comparable to FBS (xeno-containing) conditions [14]. These media-dependent observations indicate that culture-associated variability remains a limitation of hiPSC systems, independent of xeno-containing or xeno-free status.

Despite the widespread use of commercially available hiPSC media, direct quantitative comparisons of proliferation, pluripotency, and culture stability under standardized automated conditions remain limited. In addition to commercially available formulations, numerous laboratory-specific media formulations are used, compounding variability across studies. Addressing this methodological gap is essential for improving consistency in stem cell research and for developing scalable, standardized workflows suitable for translational and clinical applications [15,16]. Additionally, subtle differences in operator handling, passage timing, and feeding schedules can accumulate across passages that influence cellular stress responses, growth rates, gene expression, differentiation state, and downstream functional outputs [17]. This variability hinders reproducibility across studies and remains a major barrier to standardization in stem cell research and clinical translation [18].

Herein, we explored the use of automated tissue culture and imaging platforms paired with machine learning-based, artificial intelligence (AI) image analysis as a strategy to standardize culture monitoring. Through real-time imaging and trained analytical models, these systems enable standardization of feeding schedules, passaging intervals, and endpoint assessments, improving phenotypic consistency relative to conventional culture methods [19,20,21].

## 2. Methods

### 2.1. hiPSC Culture

Wild-type hiPSC line iP11N (ALSTEM Cell Advancements, Richmond, CA, USA) were maintained on hESC-qualified Matrigel (Corning, CLS354277-1EA, Corning, NY, USA) in Costar^®^ 6-well clear tissue culture-treated plates (Corning, 3506, Corning, NY, USA) and Costar^®^ 24-well clear tissue culture-treated plates (Corning, 3527, Corning, NY, USA). Cells were cultured in either mTeSR+ (STEMCELL Technologies, 100-0276, Vancouver, BC, Canada) or StemFlex medium (Thermo Fisher Scientific, A3349401, Waltham, MA, USA) and maintained at 37 °C in a humidified incubator with 5% CO_2_.

Cells were passaged using ReLeSR (STEMCELL Technologies, 100-0483, Vancouver, BC, Canada) according to manufacturer guidelines with minor modifications. Briefly, spent media was aspirated, and wells were rinsed with DMEM/F-12 (Thermo Fisher Scientific, 11320033, Waltham, MA, USA). ReLeSR (500 µL per well of a 6-well plate) was applied and removed within 1 min, leaving a thin residual film. Plates were incubated at room temperature for 3–7 min until colonies began to detach.

Colonies were mechanically dislodged using a wide-bore pipette tip in pre-warmed culture media and transferred at split ratios ranging from 1:3 to 1:12 onto Matrigel-coated plates containing mTeSR+ or StemFlex medium. Plates were returned to a 37 °C incubator and gently agitated to ensure even distribution of cell aggregates. Cells were left undisturbed for at least 24 h to allow attachment and recovery. Media changes were performed every other day by aspirating spent media and replacing it with fresh medium (2 mL per well for 6-well plates; 0.5 mL per well for 24-well plates) unless otherwise specified.

### 2.2. StemFlex Acclimation

One vial of frozen iP11N hiPSCs, previously acclimated to mTeSR+, was thawed in mTeSR+ in two wells of a 6-well plate. After 48 h, one well was maintained in mTeSR+, while the second well was transitioned to StemFlex over four days using a stepwise serial dilution: 75% mTeSR+25% StemFlex, followed by 50% mTeSR+50% StemFlex, followed by 25% mTeSR+75% StemFlex, before reaching 100% StemFlex. These cells were then cultured exclusively in StemFlex medium for three additional passages before downstream experiments. Colony morphology, growth rate, and overall cell health were assessed throughout the acclimation period.

### 2.3. CellXpress.ai Imaging, Media Changing, and Analysis

hiPSCs were maintained, imaged and analyzed on the CellXpress.ai (Molecular Devices, San Jose, CA, USA). Cells were first seeded onto 6-well or 24-well Costar plates that were precoated with hESC-qualified Matrigel according to the manufacturer’s instructions at 120,000 cells per well. Brightfield images were acquired every 24 h on the CellXpress.ai via transmitted Light channel using a 4X-0.2NA objective standard. Multiple sites were imaged for each well with an exposure time of 2.5 ms and a focus offset of −69 um. Cells were fed every 48 h via media exchange using mTeSR+ or StemFlex media. Media exchanges were performed on the CellXpress.ai liquid handler using 1000 uL CO-RE II disposable tips (Hamilton 235940, Reno, NV, USA). Used media was aspirated at a rate of 150 uL/s from a height of 1mm above the surface of the plate and disposed of. Fresh media was warmed to room temperature and dispensed into each well at a rate of 100 uL/s at a height of 3 mm above the surface of the plate. Plates were tilted during media aspiration and dispensing, and new tips were used for each well.

Image analysis used two custom deep-learning segmentation models trained in the SINAP module of IN Carta [v2.8] (Molecular Devices, San Jose, CA, USA): Differentiated. C, for differentiated cells present within the stem-cell culture, and StemCells.c, for hiPSC colonies. SINAP models are U-Net-style encoder–decoder convolutional networks with a ResNet101 encoder retrained with nuclei and cell images; the architecture is proprietary, and this description was provided by the vendor. Both models were fine-tuned from the respective base model on manually annotated 4× transmitted-light fields of view, across iterative rounds of annotation and retraining. The differentiated-cell model was developed over five rounds of training using 21 unique annotated fields; the final model was used for all reported analyses. The hiPSC model was developed in two phases—initial training on 6 fields, followed by refinement on 16 additional fields—and the final model was used for all reported analyses with images analyzed under earlier model versions reanalyzed using the final model. The IN Carta segmentation model was trained on individual fields of view. Longitudinal growth analysis aggregated all fields of view within a well at each timepoint into a single data point representing that well. Annotated training sets and model files are available upon request. As a negative control, both models were applied to empty-well fields; no pixels were classified as target across 6 fields, confirming that background artifacts and debris were not misclassified as cells. Model training was considered complete when visual inspection of segmentation overlays showed no residual false-positive regions and satisfactory agreement with expert annotation of cell boundaries, evaluated qualitatively during the training process. Confluency was calculated as the fraction of field area assigned to the target class.

All imaging, model training, and quantitative analyses were conducted under blinded conditions using randomized plate identifiers generated by the CellXpress.ai, which were unblinded by the software after data analysis.

### 2.4. Immunocytochemistry (ICC)

Cells were plated onto 12 mm width, 1.5 mm thickness glass coverslips in 24-well plates coated with hESC-qualified Matrigel. Cells were cultured for 3–7 days at 37 °C until colonies reached approximately 30–70% confluency. Cells were fixed with 4% paraformaldehyde for 20 min at room temperature, followed by three washes with Dulbecco’s phosphate-buffered saline (dPBS; Gibco, 14190-136, Waltham, MA, USA).

Cells were permeabilized and blocked using a solution of 20% normal goat serum (NGS; Thermo Fisher Scientific, 10000C) in 0.3% Triton X-100 (Sigma, T9284, St. Louis, MO, USA) in dPBS for 1 h at room temperature. Primary antibodies were diluted in 20% NGS in 0.3% Triton X-100 PBS blocking solution (dilutions in Table 1) and incubated overnight at 4 °C. Following primary antibody incubation, cells were washed three times with dPBS for five minutes each. Fluorophore-conjugated secondary antibodies were diluted 1:1000 in blocking solution and applied for 2 h at room temperature in the dark. Cells were then incubated with DAPI for five minutes and underwent 3 dPBS washes. Coverslips were mounted onto Superfrost Plus microscope slides (Fisherbrand, 12-550-15, Waltham, MA, USA) using ProLong™ Glass Antifade Mounting Medium (Thermo Fisher, P36980) and cured for 72 h in a dark box with adequate airflow at room temperature before imaging.

All images within each experimental condition were acquired using identical exposure settings on a KEYENCE BZ-X800 benchtop fluorescence microscope (KEYENCE Corporation of America, Itasca, IL, USA). Image processing, including channel merging and adjustments, was performed using Keyence BZ-X800 software (version 1.1.30.19). Antibody details and exposure parameters are provided in Table 1 and Table 2, respectively.

ICC image acquisition was conducted under blinded conditions using randomized sample identifiers, ensuring that experimental group identity was concealed throughout imaging.

Quantitative analysis of ICC images was performed using FIJI [v2.16.0/1.54P] (ImageJ, National Institutes of Health, Bethesda, MD, USA, version 2.16.0/1.54P). Images acquired at 20× magnification were analyzed using a consistent workflow across both culture conditions. Images were segmented using Otsu automatic thresholding followed by particle analysis. Four wells were analyzed per culture condition (StemFlex and mTeSR+). Particle count and percentage area (%Area) were recorded to assess cell-associated density and area coverage.

### 2.5. RNA Isolation

Total RNA was isolated using the RNeasy Mini Kit (Qiagen, 74104, Germantown, MD, USA) according to the manufacturer’s instructions. Briefly, cells were lysed in a lysis buffer containing β-mercaptoethanol, homogenized, and subjected to on-column DNase digestion. RNA was purified using silica membrane spin columns and eluted in 35 µL nuclease-free water.

RNA concentration and purity were measured using a NanoDrop One spectrophotometer (Thermo Fisher Scientific, 13-400-518). RNA integrity and yield were assessed prior to downstream applications, and only samples meeting quality and concentration criteria were used for reverse transcription.

### 2.6. Reverse Transcription and cDNA Synthesis

cDNA was synthesized using the High-Capacity cDNA Reverse Transcription Kit (Thermo Fisher Scientific, 4368814) according to the manufacturer’s instructions. RNA input was standardized across samples, with 1000 ng of total RNA used per 20 µL reaction. For samples requiring increased input, reaction volumes were scaled proportionally. Reverse transcription reactions were performed using MultiScribe reverse transcriptase and random primers (Thermo Fisher Scientific, 4368814) in a thermal cycler under manufacturer-recommended conditions. PCR setup was as follows: Step 1 was performed at 25 °C for 10 min, step 2 at 37 °C for 120 min, step 3 at 85 °C for 5 min, and step 4 at 4 °C with an infinite hold.

### 2.7. Quantitative Polymerase Chain Reaction

Quantitative polymerase chain reaction (qPCR) was performed using SsoAdvanced Universal SYBR Green Supermix (Bio-Rad, 1725270, Hercules, CA, USA). Reactions were prepared in 10 µL volumes using gene-specific primers (Table 3) and 1 µL cDNA template. No-template molecular grade water (Fisher Bioreagents, BP2819-1, Waltham, MA, USA) controls were utilized in duplicates or triplicates for each assay. Reactions were plated in 96-well plates (USA Scientific, 1402-9700, Ocala, FL, USA) and spun at 100× *g* for 1 min before being run on a QuantStudio 3 Real-Time PCR System (Thermo Fisher Scientific, Waltham, MA, USA).

The Excel report containing quantification cycle (Cq) values, melt curve values, and all quality control metrics produced was exported in the Design and Analysis 2 QuantStudio software (Thermo Fisher Scientific, Waltham, MA, USA, version 2.8.0). Relative gene expression was calculated using the comparative Cq (ΔΔCq) method. For each sample, target gene expression was normalized to the endogenous control gene β-ACTIN by calculating ΔCq (Cq target − Cq β-ACTIN). ΔCq values were then normalized to the average ΔCq of the mTeSR+ condition to generate ΔΔCq values. Relative expression levels were calculated as 2ΔΔCq and are reported as fold change relative to mTeSR+ controls.

**Figure 1:**
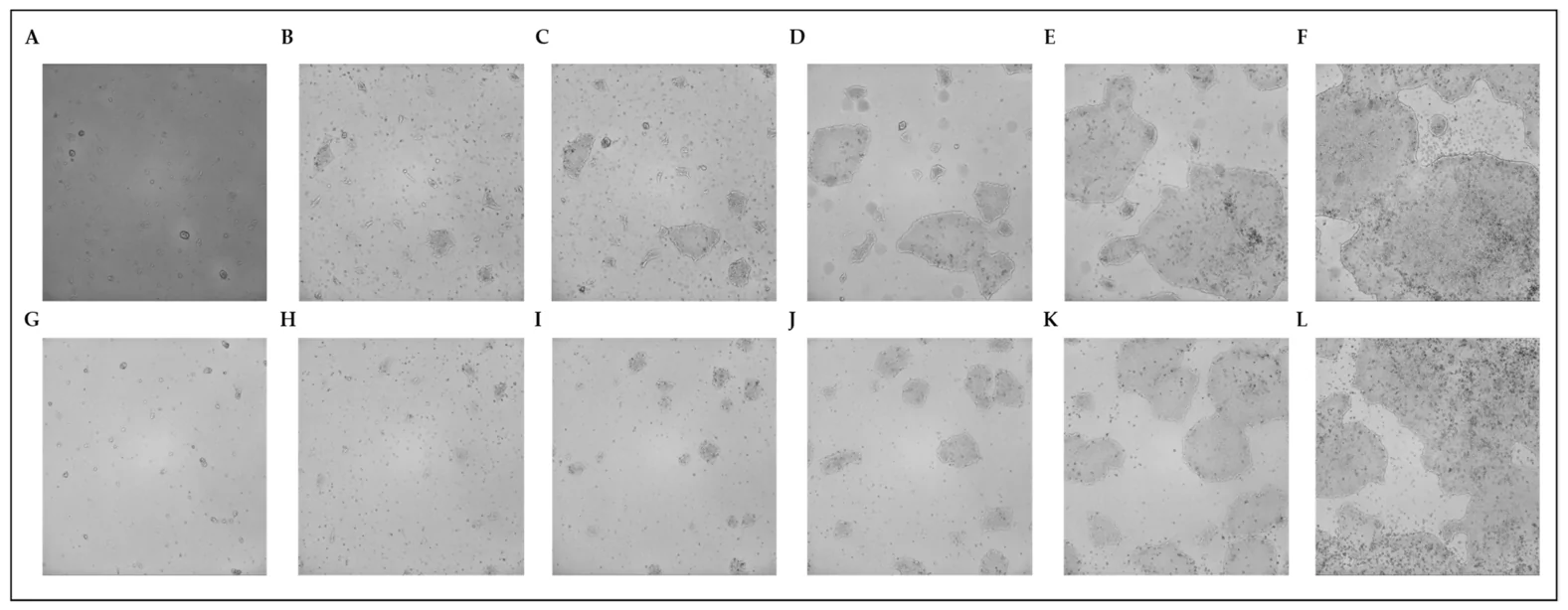
Time-lapse bright-field imaging of human pluripotent stem cells cultured in StemFlex or mTeSR+ medium. Representative illustrative images (4× magnification, 5 × 5 stitched acquisition) were acquired daily from Well A1 (Image 2 of 25) using the CellXpress.ai automated imaging platform over a 120-h imaging period. (**A**–**F**) StemFlex: (**A**) 0 h, (**B**) 24 h, (**C**) 48 h, (**D**) 72 h, (**E**) 96 h, and (**F**) 120 h. (**G**–**L**) mTeSR+: (**G**) 0 h, (**H**) 24 h, (**I**) 48 h, (**J**) 72 h, (**K**) 96 h, and (**L**) 120 h. Images represent one of 25 fields of view captured per well at each timepoint. A routine media change was performed between images from 48 and 72 h and 96 and 120 h. Local contrast enhancement was applied equally across all panels in FIJI for visualization purposes only.

**Figure 2:**
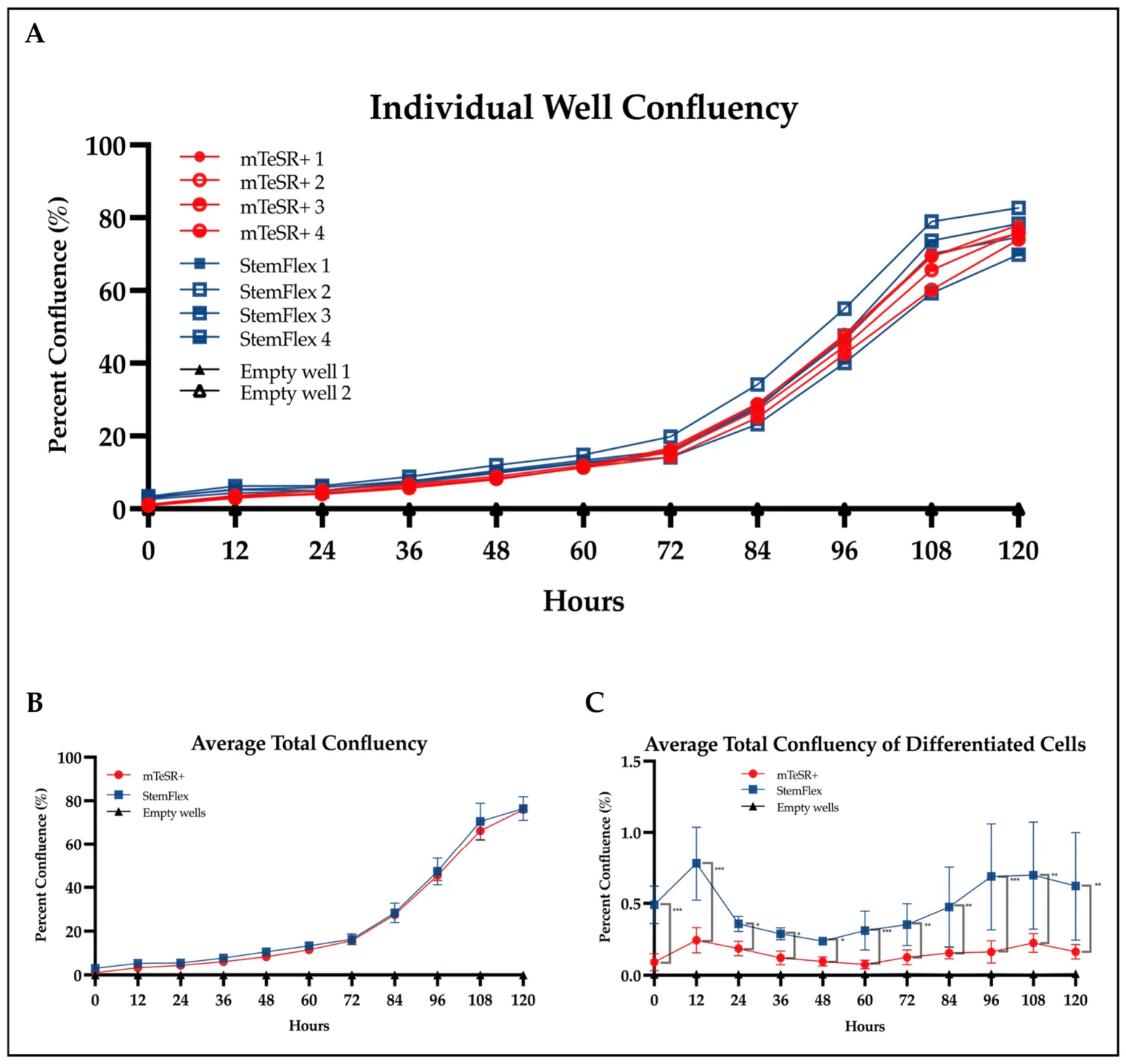
Quantitative analysis of iP11N hiPSC growth kinetics in StemFlex and mTeSR+ medium measured by automated imaging. iP11N cells were cultured in mTeSR+ (red circles) or StemFlex (blue squares) medium and imaged every 12 h over 120 h using the CellXpress.ai automated imaging system at 4× magnification (5 × 5 tiled array, 150 images per plate per timepoint). Machine learning-based segmentation models were applied in IN Carta to extract quantitative metrics. (**A**) Individual well total confluence trajectories for mTeSR+ (wells 1–4), StemFlex (wells 1–4), and empty control wells (wells 1–2). (**B**) Mean total confluency percentage over time. (**C**) Mean total percent area fraction of differentiated cells over time. Data in (**B**,**C**) are presented as mean ± SD. Empty wells served as negative controls and maintained zero confluence and zero total area across all timepoints. No statistically significant differences in total growth rate or doubling time were observed between conditions (**A**,**B**). Statistical comparisons of the differentiated cell area fraction (**C**) were performed using a linear mixed-effects model with medium, time, and their interaction as fixed effects and well as a random effect, to account for repeated measurements from the same wells. Pairwise comparisons between StemFlex and mTeSR+ at each timepoint were derived from the fitted model and corrected for multiple comparisons using the Holm–Bonferroni method (* *p* < 0.05, ** *p* < 0.01, *** *p* < 0.001). StemFlex-maintained cultures exhibited significantly greater differentiated cell area fraction than mTeSR+-maintained cultures at every imaging timepoint (t = 0 h: *p* < 0.001 ***; t = 12 h: *p* < 0.001 ***; t = 24 h: *p* = 0.038 *; t = 36 h: *p* = 0.012 *; t = 48 h: *p* = 0.012 *; t = 60 h: *p* < 0.001 ***; t = 72 h: *p* = 0.009 **; t = 84 h: *p* = 0.008 **; t = 96 h: *p* < 0.001 ***; t = 108 h: *p* = 0.003 **; t = 120 h: *p* = 0.001 **) The empty wells are present in all 3 parts of this figure. (**A**) is individualized wells. (**B**,**C**) are averaged.

**Figure 3:**
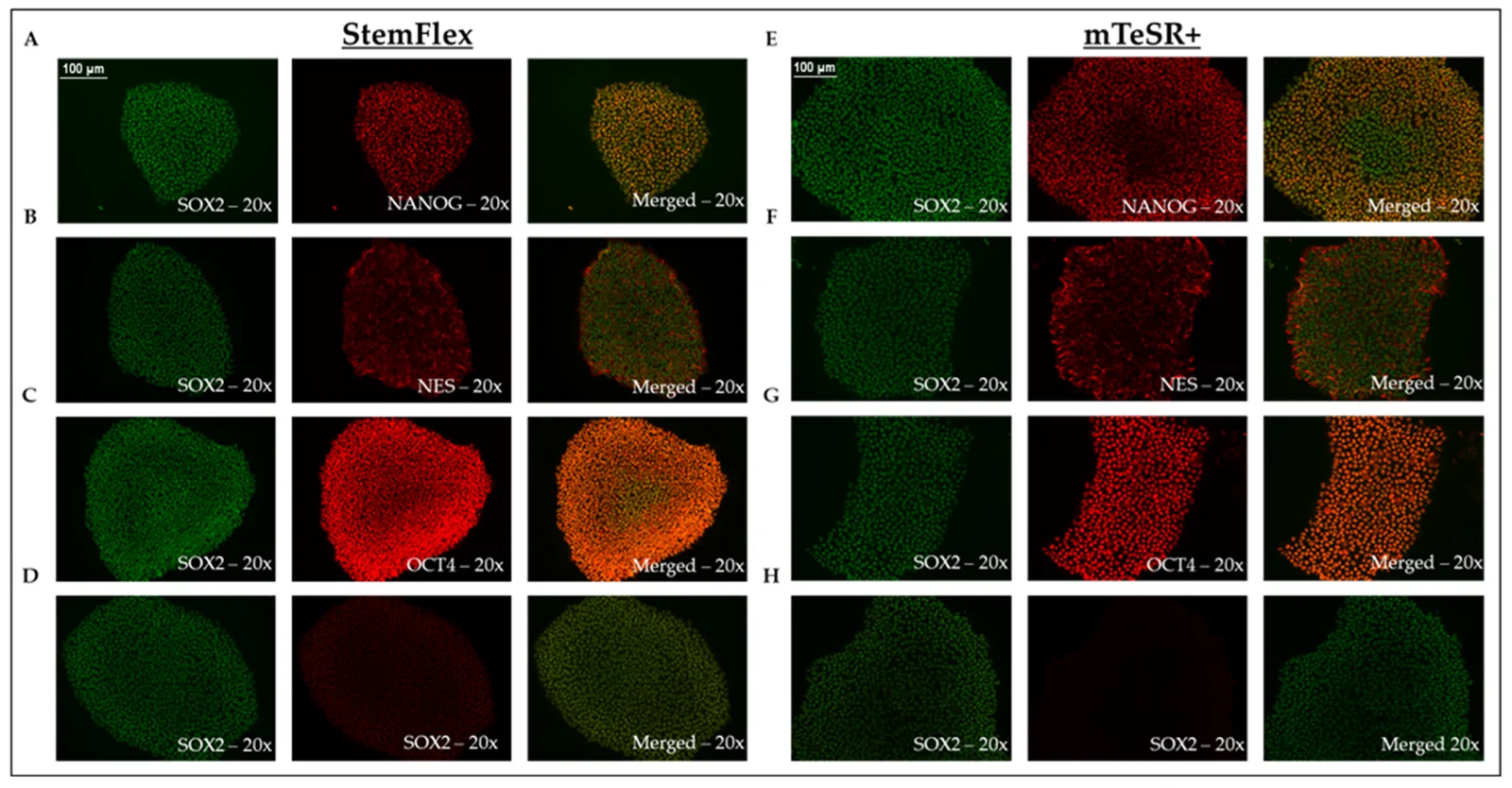
Immunocytochemistry (ICC) of pluripotency and neural markers in iP11N cells cultured in StemFlex or mTeSR+ medium. Representative fluorescence images were obtained from four independent wells per culture condition (StemFlex and mTeSR+) in a 24-well plate and acquired at 20× magnification. SOX2 (green) was used as a co-stain in all panels. (**A**) StemFlex: SOX2 (green), NANOG (red), and merged. (**B**) StemFlex: SOX2 (green), NESTIN (NES; red), and merged. (**C**) StemFlex: SOX2 (green), POU5F1/OCT4 (OCT4; red), and merged. (**D**) StemFlex: SOX2 (green), SOX2 (red), and merged. (**E**) mTeSR+: SOX2 (green), NANOG (red), and merged. (**F**) mTeSR+: SOX2 (green), NES (red), and merged. (**G**) mTeSR+: SOX2 (green), OCT4 (red), and merged. (**H**) mTeSR+: SOX2 (green), SOX2 (red), and merged. Antibody details and incubation times are listed in Table 1 and Table 2, respectively.

**Figure 4:**
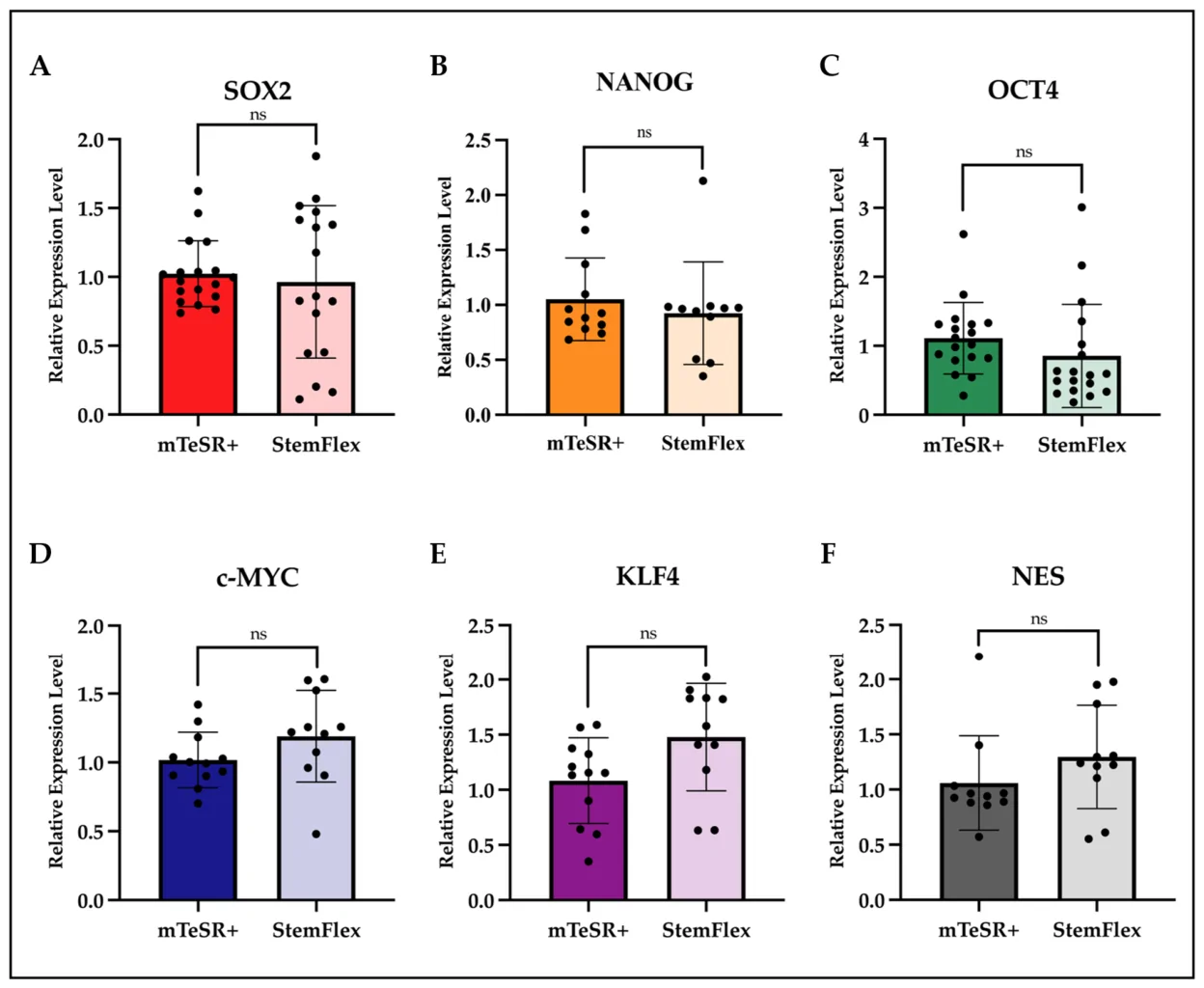
Quantitative RT-PCR analysis of pluripotency and neural marker gene expression in iP11N cells cultured in StemFlex or mTeSR+ medium following automated culture. Relative gene expression levels were normalized to a standardized amount of cDNA input. Data are presented as mean ± SD with all individual data points shown. Statistical comparisons were performed using the Mann–Whitney U test, with Holm–Bonferroni correction applied across all six genes. (**A**) *SOX2* expression (mTeSR+ *n* = 18, StemFlex *n* = 17; adjusted *p* = 1.000, not significant (ns)). (**B**) *NANOG* expression (mTeSR+ *n* = 12, StemFlex *n* = 12; adjusted *p* = 1.000, ns). (**C**) *OCT4* expression (mTeSR+ *n* = 18, StemFlex *n* = 18; adjusted *p* = 0.218, ns). (**D**) *c-MYC* expression (mTeSR+ *n* = 12, StemFlex *n* = 11; adjusted *p* = 0.363, ns). (**E**) *KLF4* expression (mTeSR+ *n* = 12, StemFlex *n* = 11; adjusted *p* = 0.161, ns). (**F**) *NES* expression (mTeSR+ *n* = 11, StemFlex *n* = 11; adjusted *p* = 0.363, ns). No statistically significant differences in gene expression were detected between culture conditions after correction for multiple comparisons. Each individual datapoint is illustrated as a black dot.

For each culture condition (mTeSR+, StemFlex), 4 independent wells were maintained, of which 3 were harvested for RNA isolation, constituting 3 independent biological replicates per condition. Technical qPCR replicates were run for each biological replicate to utilize available plate capacity in two runs (SOX2, OCT4: triplicate; NANOG, c-MYC, KLF4: triplicate), with one technical replicate omitted for a subset of biological replicates due to plate capacity or quality control exclusion. Reported sample sizes (*n*) in Figure 1 reflects a representative time-lapse of one biological sample per media condition. Figure 2, Figure 3 and Figure 4 contain both biological and technical replicates.

### 2.8. qPCR Primers

All primer sequences were based on human genes or transcription factors and obtained from OriGene (OriGene Technologies, Rockville, MD, USA). Primer sequences were ordered from IDT (Integrated DNA Technologies, Coralville, IA, USA) and utilized at 10 µM in sterile pH 8 TE buffer.

### 2.9. Image-Based Doubling Time Analysis

Cell proliferation was quantified using longitudinal live-cell imaging combined with image-based segmentation, where total cell area was used as a surrogate for cell number. For each well, total cell area was measured at 12-h intervals over a 120-h imaging period. Timepoints were converted to elapsed time in hours relative to the first acquisition (t_0_ = 0 h). Quantitative analysis was restricted to timepoints corresponding to logarithmic growth (60–108 h), prior to the onset of confluence-associated growth arrest. Linear regression of natural log (ln)-transformed total cell area against time within this window showed strong linearity for both conditions (R^2^ = 0.986 for mTeSR+, 0.953 for StemFlex), supporting selection of the 60–108 h window as representative of exponential growth (Supplementary Figure S1). For each well, total cell area measurements collected during the exponential growth phase (t = 60, 72, 84, 96, and 108 h) were ln-transformed and modeled using linear regression based on the exponential growth equation:

A(t) = A_0_e^(kt)^

where A(t) represents total cell area at time t, A_0_ denotes the area at t_0_, and k is the growth rate constant (h^−1^). Linear regression was performed on ln-transformed area values as a function of time using ordinary least squares, and growth rate constants were obtained from the slope of the fitted regression line for each well. Doubling times were calculated as

Td = ln(2)/k

and summarized across biological replicates as mean ± standard deviation.

Early and late timepoints, during which growth deviated from exponential behavior and approached a plateau, were excluded from model fitting and used only for visualization.

### 2.10. Image Contrast Enhancement

Representative brightfield images were processed for visualization using the Contrast Limited Adaptive Histogram Equalization (CLAHE) function in FIJI [v2.16.0/1.54P] (ImageJ, National Institutes of Health, Bethesda, MD, USA, version 2.16.0/1.54P). Images were processed using the Enhance Local Contrast plugin (Process → Enhance Local Contrast) with default parameters applied uniformly across all images. Contrast enhancement was performed solely for visualization purposes and was not applied to images used for quantitative analysis.

### 2.11. Statistical Analysis

All statistical analyses were performed using Python (SciPy; www.scipy.org (accessed on 17 August 2026), version 3.10.7) or GraphPad Prism (GraphPad Software, San Diego, CA, USA, version 10.6.1 (892)) as appropriate. For RT-qPCR gene expression comparisons between mTeSR+ and StemFlex conditions, data were first assessed for normality. Several genes did not meet the assumption of a Gaussian distribution and comparisons between conditions were performed using the Mann–Whitney U test in GraphPad Prism (GraphPad Software, San Diego, CA, USA, version 10.6.1 (892)). Resulting *p*-values across the six genes assessed were corrected for multiple comparisons using the Holm–Bonferroni method. For cell proliferation analyses, growth rate constants were derived from ordinary least squares linear regression of ln-transformed cell area values across the exponential growth phase (t = 60–108 h), and doubling times between culture conditions were compared using an unpaired two-tailed Student’s *t*-test in Python (SciPy; www.scipy.org (accessed on 17 August 2026), version 3.10.7). For image-based differentiated cell area fraction analyses, we used a linear mixed-effects model. Medium and time were included as fixed effects, along with their interaction, to test the two culture conditions over time. Each well was measured repeatedly at identical areas at 11 timepoints and well identity was included as a random effect, allowing the model to account for repeated measurements from the same well. Pairwise comparisons between StemFlex and mTeSR+ at each timepoint were derived from the fitted model, with correction for multiple comparisons using the Holm–Bonferroni method. Statistical significance was defined as *p* < 0.05, with significance levels denoted as * *p* < 0.05, ** *p* < 0.01, and *** *p* < 0.001.

## 3. Results

### 3.1. hiPSCs Exhibit Comparable Morphology and Growth Phenotype

Longitudinal live-cell imaging using the CellXpress.ai system revealed no detectable differences in colony morphology between culture conditions or media (Figure 1A–F, StemFlex; G-L, mTeSR+). Across the 120-h imaging period, iP11N hiPSCs exhibited similar colony shape, edge definition, and spatial organization, with no observable morphological abnormalities. Colonies maintained a characteristic compact morphology with smooth borders and uniform appearance within 24 h of passage (Figure 1B, StemFlex; 1H, mTeSR+).

### 3.2. Comparable Cell Growth Rates Across Culture Conditions

Automated growth analysis using the CellXpress.ai system showed no detectable differences in cell proliferation between mTeSR+ and StemFlex wells (Figure 2A). Percent confluence (Figure 2B) exhibited identical growth trajectories across conditions throughout the imaging period.

Quantitative analysis of growth rate constants and doubling times derived from exponential-phase cell area measurements (t = 60–108 h) revealed no statistically significant difference between culture conditions. mTeSR+-maintained cells exhibited a mean doubling time of 18.25 ± 0.38 h compared to 19.00 ± 0.89 h for StemFlex-maintained cells (Student’s two-tailed t-test, *p* = 0.176), indicating comparable proliferative capacity across media formulations under the tested conditions. Linear regression of ln-transformed cell area against time within the 60–108 h window showed strong linearity for both conditions (R^2^ = 0.986 for mTeSR+, 0.953 for StemFlex), supporting selection of this window as representative of exponential growth (Supplementary Figure S1). Empty control wells consistently remained at/around zero total area and zero percent confluence across all timepoints, supporting the specificity of the image-based segmentation and analysis pipeline.

Quantitative analysis of the differentiated cell area fraction across eleven imaging timepoints revealed that StemFlex-maintained cultures consistently exhibited a significantly greater differentiated cell area fraction (Figure 2C) compared to mTeSR+-maintained cultures. To account for repeated measurements collected from the same wells over time, differences between conditions were assessed using a linear mixed-effects model with medium, time, and their interaction as fixed effects and well as a random effect. Pairwise comparisons at each timepoint were derived from the fitted model and corrected for multiple comparisons using the Holm–Bonferroni method. StemFlex-maintained cultures exhibited a significantly greater differentiated cell area fraction than mTeSR+-maintained cultures at every imaging timepoint (t = 0 h: *p* < 0.001; t = 12 h: *p* < 0.001; t = 24 h: *p* = 0.038; t = 36 h: *p* = 0.012; t = 48 h: *p* = 0.012; t = 60 h: *p* < 0.001; t = 72 h: *p* = 0.009; t = 84 h: *p* = 0.008; t = 96 h: *p* < 0.001; t = 108 h: *p* = 0.003; t = 120 h: *p* = 0.001). At peak divergence (t = 108 h), StemFlex cultures displayed a mean differentiated cell area fraction of 0.919 ± 0.595% compared to 0.342 ± 0.267% in mTeSR+ cultures. Together, these data demonstrate that automated image-based quantification, combined with a repeated-measures statistical framework, identified a consistent, statistically significant, yet sub-percent difference in differentiated cell area fraction between culture conditions across the entire 120-h imaging period, a magnitude that would unlikely be detectable by conventional endpoint assays.

### 3.3. Immunocytochemistry Demonstrates Comparable Pluripotency Marker Expression Across Conditions

Immunocytochemical (ICC) analysis of iP11N hiPSCs cultured in mTeSR+ and StemFlex media cultured in 24-well plates with coverslips showed no detectable differences in the expression or localization of canonical pluripotency markers (Figure 3A–H). Staining for SOX2 (Figure 3A,D,E,H), NANOG (Figure 3A,E), NESTIN (NES; Figure 3B,F), and POU5F1/OCT4 (OCT4; Figure 3C,H) demonstrated similar signal intensity and localization patterns across conditions. Cells maintained uniform marker expression consistent with an undifferentiated state, with no evidence of marker loss, heterogeneous expression, or differentiation in either condition following one passage after automation studies. Quantitative ImageJ analysis was performed to evaluate cell density and area between culture conditions. StemFlex cultures had a mean particle count of 1012 ± 579 per field and mean area coverage of 21.88 ± 10.11%, compared with 1565 ± 428 particles per field and 28.94 ± 9.90% area coverage for mTeSR+ cultures (four wells per condition; Supplementary Figure S4). Despite the visually more compact appearance of StemFlex colonies, quantitative image analysis did not demonstrate greater particle density or area coverage relative to mTeSR+.

### 3.4. RT-qPCR Analysis Demonstrates No Significant Transcriptional Differences Between Media Conditions

RT-qPCR analysis of pluripotency-associated genes SOX2, NANOG, OCT4, and c-MYC, as well as the neural progenitor marker NES, showed no significant differences between iP11N hiPSCs cultured in mTeSR+ and StemFlex media (Mann–Whitney U test, Holm–Bonferroni-corrected *p* > 0.05 for all comparisons). KLF4 expression was nominally higher in StemFlex-cultured cells prior to correction for multiple comparisons (mTeSR+: 1.080 ± 0.364; StemFlex: 1.407 ± 0.457; uncorrected *p* = 0.027), but this difference did not remain statistically significant after Holm–Bonferroni correction across the six genes assessed (adjusted *p* = 0.161). Across all samples, relative expression levels of SOX2 (mTeSR+: 1.024 ± 0.240; StemFlex: 0.872 ± 0.493), NANOG (mTeSR+: 1.069 ± 0.371; StemFlex: 0.835 ± 0.469), OCT4 (mTeSR+: 1.097 ± 0.491; StemFlex: 0.781 ± 0.699), c-MYC (mTeSR+: 1.018 ± 0.197; StemFlex: 1.213 ± 0.338), and NES (mTeSR+: 1.063 ± 0.457; StemFlex: 1.383 ± 0.453) were not significantly different between conditions across the three biological replicates evaluated following automated culturing (Figure 4A–E).

## 4. Discussion

In this study, we evaluated iP11N hiPSCs cultured in mTeSR+ and StemFlex feeder-free media to assess growth, morphology, pluripotency, and transcriptional profiles. Cells cultured under these conditions demonstrated no significant differences in morphology or proliferation, with automated imaging confirming comparable colony structure and growth dynamics. Longitudinal live-cell imaging revealed overlapping growth trajectories and similar doubling times in both media conditions, consistent with maintenance of hiPSC self-renewal and stem cell identity (see also Supplementary Figure S2). Quantitative image-based analysis of differentiated cell area fraction identified a statistically significant elevation in spontaneous differentiation in StemFlex-maintained cultures relative to mTeSR+ across the entire 120-h imaging period (*p* < 0.05 at every timepoint assessed), with mean differentiated cell area fractions peaking at 0.919 ± 0.595% and 0.342 ± 0.267% at t = 108 h, respectively. This low detection threshold of less than 1% highlights the utility of automated image-based quantification in identifying subtle media-dependent differences in culture composition. However, the biological significance of a <1% difference in differentiated cell composition remains to be established.

Immunocytochemistry confirmed comparable expression and localization of pluripotency markers across conditions. RT-qPCR analysis showed no significant differences in expression of pluripotency-associated genes SOX2, NANOG, OCT4, c-MYC, or the progenitor-associated marker NES between media conditions following correction for multiple comparisons. KLF4 showed a nominal increase in StemFlex-cultured cells prior to correction (uncorrected *p* = 0.027) which did not remain significant after accounting for the six genes tested (adjusted *p* = 0.161).

A separate, independent RT-qPCR dataset collected on the same clonal iP11N cell line prior to automated culture (Supplementary Figure S3) showed a significant increase in both c-MYC and KLF4 expression in StemFlex-cultured cells after correction for multiple comparisons (adjusted *p* = 0.032 and *p* = 0.007, respectively). This significance contrasts with the post-automation dataset, which appears to be attributable to differences between the pre- and post-automation experimental stages rather than media composition.

Notably, both the RT-qPCR and ICC analyses were performed on cells harvested or passaged into 24 well plates (ICC) at the conclusion of the 120-h imaging period. While the differentiated cell area fraction remained significantly elevated in StemFlex-maintained cultures relative to mTeSR+ throughout the full imaging period, the magnitude of this difference was minute (sub-1% in absolute terms), likely accounting for the absence of a corresponding significant difference in RT-qPCR and immunocytochemistry in this study. Collectively, these results suggest that both mTeSR+ and StemFlex broadly support maintenance of hiPSC identity (≥99%) under the conditions tested, though StemFlex demonstrated a consistent, low-magnitude elevation (<1%) in spontaneous differentiation throughout the culture period. Automated culture and liquid handling are expected to reduce operator-dependent variability relative to manual handling, consistent with prior reports in automated cell culture systems, while simultaneously providing the sensitivity required to detect differences in differentiation propensity. As this study did not directly compare automated and manual culture in the same experimental system, the data presented should be considered an exploratory benefit of automation supported by the broader literature rather than a finding directly demonstrated by the present dataset.

In practical applications, media selection often involves consideration of cost alongside performance. At our institution, negotiated pricing discounts StemFlex to approximately 60% of the discounted price of mTeSR+. However, this pricing is institution-specific and may not generalize to other laboratories, individuals or institutions, and does not account for other cost drivers such as feeding frequency, medium volume, labor, and quality-control requirements. Given the comparable performance observed across growth, morphology, and gene expression metrics, StemFlex may represent a cost-effective alternative for large-scale or high throughput hiPSC workflows in settings with similarly favorable pricing, provided that differentiation rates are monitored and cultures are maintained with appropriate quality controls.

The findings of this study are consistent with the broader potential of automated, machine learning-guided culture systems to reduce operator-dependent variability. Subtle differences in handling, passaging, and feeding schedules can accumulate over time and influence experimental outcomes. By incorporating real-time imaging, standardized workflows, and blinded analysis, automated platforms such as CellXpress.ai improve consistency across experiments and support reproducible data generation in stem cell research.

There are several limitations to these studies. First, this study was performed using a single wild type hiPSC line (iP11N). These findings utilizing one cell line may not represent other wild type, patient-derived, or disease-specific hiPSC lines, which can exhibit distinct proliferation dynamics, metabolic states, and spontaneous differentiation at different growth states and/or in response to different media formulations. Future studies incorporating genetically diverse and disease-relevant hiPSC models will be necessary to determine the broader applicability of these findings across pluripotent stem cell systems. Second, the proprietary nature of commercial mTeSR+ and StemFlex formulations limits mechanistic interpretation of the observed phenotypic differences. Consequently, while functional sub-percent differences in differentiated cell burden were identified through automated image analysis, the molecular drivers underlying these differences could not be directly assessed. Third, the study focused on short-term maintenance, proliferation kinetics, and pluripotency-associated marker expression under standardized automated culture conditions of one month of cell culture. Long-term genomic stability, epigenetic drift, lineage-specific differentiation efficiency, and functional maturation capacity were not evaluated and remain areas for future investigation. Finally, experiments performed using the CellXpress.ai automated culture system and IN Carta image analysis platform represent one automated system and performance may vary across alternative automated systems or laboratory-specific workflows. A primary automation run was completed (Supplementary Figure S2) which informed parameters necessary to sustain initial plating density, media changes and image timelines for Figure 2.

In conclusion, these findings demonstrate that both mTeSR+ and StemFlex robustly support hiPSC morphology, pluripotency marker expression, and proliferation dynamics under the conditions tested in the iP11N wild-type stem cell line. Notably, automated image-based analysis resolved a statistically significant yet sub-percent difference in spontaneous differentiation between conditions, a distinction that would typically be undetectable by conventional molecular endpoints, underscoring the exceptional sensitivity of automated culture platforms in identifying subtle media-dependent effects. Given this comparable functional performance, StemFlex may represent a cost-effective alternative for routine and large-scale hiPSC culture applications in institutions that follow our discount model. Together, these findings support the adoption of standardized, automated workflows to improve reproducibility, scalability, and analytical resolution in hiPSC culture systems.

## Figures and Tables

**Table 1:** Primary and secondary antibodies used for immunofluorescence staining.

Antibody Name	Antibody Catalog #	Lot	Concentration	Company	Dilution
POU5F1/OCT4 Rabbit PolyAB	II263-I-AP	00I32723	750 ug/mL	Proteintech	1:500
NANOG Rabbit PolyAB	I4295-1-AP	00I26464	750 ug/mL	Proteintech	1:500
NESTIN (NES) Rabbit PolyAB	29285-I-AP	00095I72	900 ug/mL	Proteintech	1:500
SOX2 Mouse McAb	664II-I-Ig	I004I983	2000 ug/mL	Proteintech	1:500
SOX2 Rabbit PolyAb	09-2024	2422	500 ug/mL	Stemgent	1:500
488 goat anti mouse IgG (H+L)	A11001	1484573	N/A	Life Technologies	1:1000
555 goat anti rabbit IgG (H+L)	A21428	1391902	N/A	Life Technologies	1:1000

**Table 2:** Imaging channel settings and exposure times used for fluorescence microscopy.

Channel	DAPI	eGFP	Tritc
Brightness/exposure time	1/80 s	1/5 s	1/5 s
Brightness/exposure time standard abbreviation	12.5 ms	200 ms	200 ms

**Table 3:** qPCR primers utilized.

Name	Forward Sequence	Reverse Sequence	Concentration
Human B-ACTIN	CACCATTGGCAATGAGCGGTTC	AGGTCTTTGCGGATGTCCACGT	10 µM
KLF4	CATCTCAAGGCACACCTGCGAA	TCGGTCGCATTTTTGGCACTGG	10 µM
SOX2	GCTACAGCATGATGCAGGACCA	TCTGCGAGCTGGTCATGGAGTT	10 µM
c-MYC	CCTGGTGCTCCATGAGGAGAC	CAGACTCTGACCTTTTGCCAGG	10 µM
NANOG	CTCCAACATCCTGAACCTCAGC	CGTCACACCATTGCTATTCTTCG	10 µM
POU5F1/OCT4	CCTGAAGCAGAAGAGGATCACC	AAAGCGGCAGATGGTCGTTTGG	10 µM
NESTIN (NES)	TCAAGATGTCCCTCAGCCTGGA	AAGCTGAGGGAAGTCTTGGAGC	10 µM

## Data Availability

The original contributions presented in this study are included in the article/Supplementary Material. Further inquiries can be directed to the corresponding author.

## Supplementary Materials

The following supporting information can be downloaded at: https://www.mdpi.com/article/10.3390/cells15171533/s1, Supplementary Figure S1. Diagnostic assessment of linear regression fit supporting selection of the exponential growth window. Total cell area (µm^2^) was log-transformed and plotted against time for each biological replicate well cultured in mTeSR+ (red) or StemFlex (blue) medium. Ordinary least squares linear regression was fit to three candidate time windows to evaluate goodness of fit. For each panel, the fitted regression line is displayed as a solid line within the fitted window (shaded region) and extrapolated outside this window for visual reference (dashed line). Individual markers indicate distinct biological replicate wells (A-1, A-2, B-1, B-2). (A) Full 0-120 h range: mTeSR+ (R^2^ = 0.974, slope = 0.0334 h^−1^, doubling time = 20.73 h); StemFlex (R^2^ = 0.971, slope = 0.0272 h^−1^, doubling time = 25.47 h). (B) 60-108 h window: mTeSR+ (R^2^ = 0.986, slope = 0.0380 h^−1^, doubling time = 18.25 h); StemFlex (R^2^ = 0.953, slope = 0.0365 h^−1^, doubling time = 18.96 h). (C) 12-60 h window: mTeSR+ (R^2^ = 0.978, slope = 0.0261 h^−1^, doubling time = 26.52 h); StemFlex (R^2^ = 0.898, slope = 0.0209 h^−1^, doubling time = 33.16 h); Supplementary Figure S2. Secondary quantitative analysis of hiPSC growth kinetics and differentiated cell burden in mTeSR+ and StemFlex media measured by automated live-cell imaging. iP11N cells were maintained in mTeSR+ (red, circles) or StemFlex (blue, squares) medium and imaged every 12 hours over 108 hours using the CellXpress.ai automated imaging platform at 4× magnification. Machine learning–based segmentation models were applied in IN Carta to quantify cellular growth and differentiation-associated metrics. (A) Individual well confluence trajectories for biological replicates cultured in mTeSR+ and StemFlex conditions. (B) Total confluence (%) over time. (C) Mean total confluence (%) of differentiated cells over time. Data are presented as mean ± SD; Supplementary Figure S3. Quantitative RT-qPCR analysis of pluripotency and neural marker gene expression in iP11N hiPSCs cultured in StemFlex or mTeSR+ medium prior to automated culture. Relative gene expression levels were normalized to a standardized amount of cDNA input. Data are presented as mean ± SD with individual data points shown. As several genes did not meet the assumption of a Gaussian distribution, statistical comparisons were performed using the Mann-Whitney U test, with resulting p-values corrected for multiple comparisons across the six genes assessed using the Holm-Bonferroni method; ns = not significant. (A) SOX2 expression (mTeSR+ n = 24, StemFlex n = 24; adjusted *p* = 1.000, ns). (B) NANOG expression (mTeSR+ n = 17, StemFlex n = 16; adjusted *p* = 0.781, ns). (C) OCT4 expression (mTeSR+ n = 24, StemFlex n = 24; adjusted *p* = 1.000, ns). (D) c-MYC expression (mTeSR+ n = 21, StemFlex n = 25; adjusted *p* = 0.032, *). (E) KLF4 expression (mTeSR+ n = 24, StemFlex n = 24; adjusted *p* = 0.007, **). (F) NES expression (mTeSR+ n = 5, StemFlex n = 5; adjusted *p* = 0.603, ns). Statistically significant differences in expression were detected for c-MYC and KLF4, with all other genes remaining non-significant between culture conditions after correction for multiple comparisons; Supplementary Figure S4. Quantitative ImageJ analysis of SOX2-positive particle count and area coverage in iP11N hiPSCs cultured in mTeSR+ and StemFlex media. Representative 20× ICC images from four wells per culture condition were analyzed using ImageJ. The SOX2/GFP channel was segmented using Otsu automatic thresholding followed by particle analysis. (A) SOX2-positive particle count per field and (B) percentage area (%Area) were quantified for mTeSR+ and StemFlex cultures. Data are presented as individual well values with mean ± SD (n = 4 wells per condition). StemFlex cultures showed a mean particle count of 1,012 ± 579 per field and mean area coverage of 21.88 ± 10.11%, compared with 1565 ± 428 particles per field and 28.94 ± 9.90% area coverage for mTeSR+ cultures.
