# Supplementary material for: Machine Learning-Driven Automated Culture Reveals Similar Performance of mTeSR+ and StemFlex in Human iP11N Pluripotent Stem Cells
Source: Cells. 2026 Aug 25;15(17):1533. doi: 10.3390/cells15171533 (PMC13565254; doi:10.3390/cells15171533)
Supplement: Supplementary file 1 [file cells-15-01533-s001.zip › Supplementary figures.pdf]

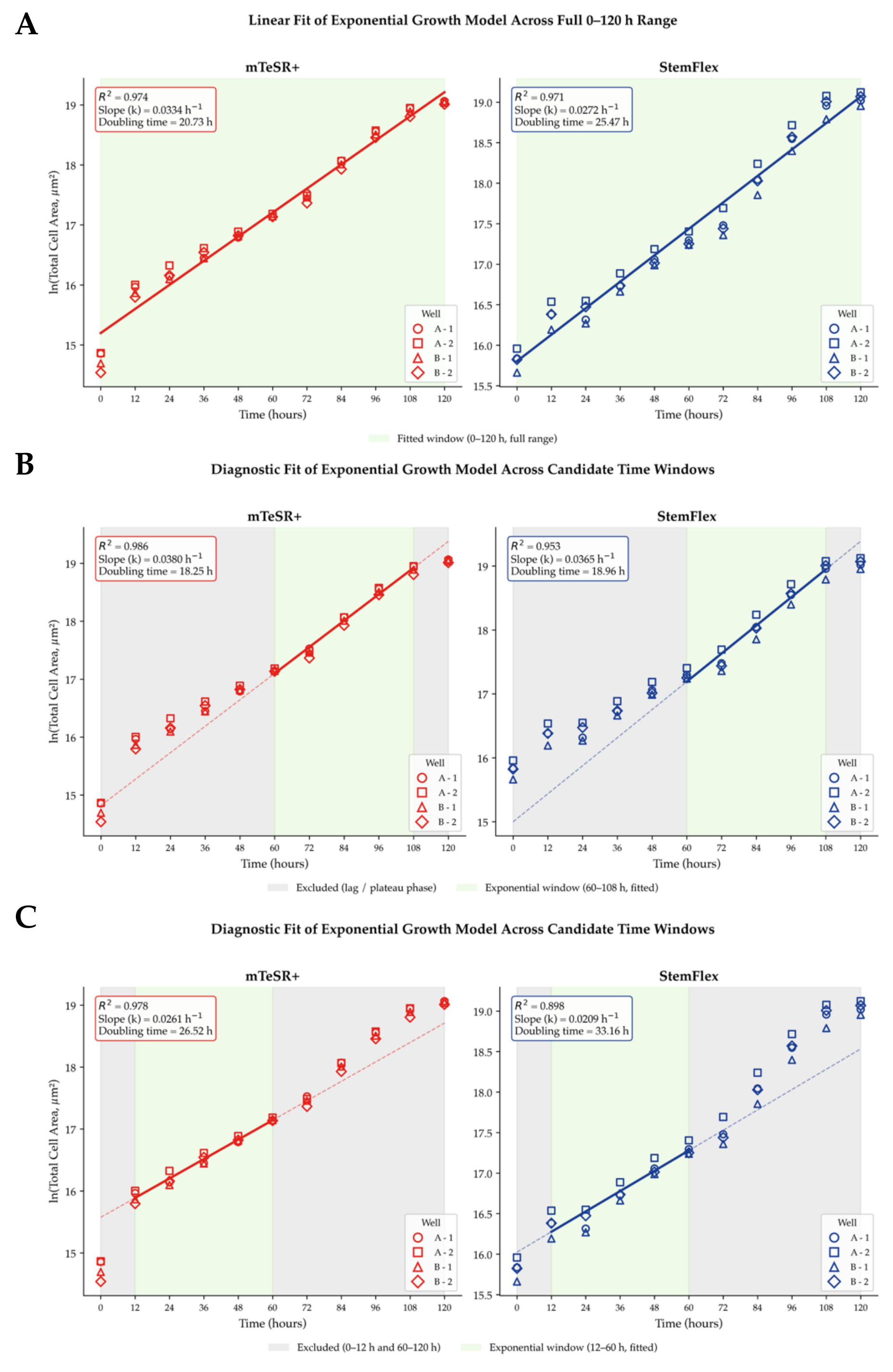

Supplementary Figure S1. Diagnostic assessment of linear regression fit supporting selection of the exponential growth window. Total cell area (μm²) was log-transformed and plotted against time for each biological replicate well cultured in mTeSR+ (red) or StemFlex (blue) medium. Ordinary least squares linear regression was fit to three candidate time windows to evaluate goodness of fit. For each panel, the fitted regression line is displayed as a solid line within the fitted window (shaded region) and extrapolated outside this window for visual reference (dashed line). Individual markers indicate distinct biological replicate wells (A-1, A-2, B-1, B-2). (A) Full 0-120 h range: mTeSR+ (R^2 = 0.974, slope = 0.0334 h^{-1}, doubling time = 20.73 h); StemFlex (R^2 = 0.971, slope = 0.0272 h^{-1}, doubling time = 25.47 h). (B) 60-108 h window: mTeSR+ (R^2 = 0.986, slope = 0.0380 h^{-1}, doubling time = 18.25 h); StemFlex (R^2 = 0.953, slope = 0.0365 h^{-1}, doubling time = 18.96 h). (C) 12-60 h window: mTeSR+ (R^2 = 0.978, slope = 0.0261 h^{-1}, doubling time = 26.52 h); StemFlex (R^2 = 0.898, slope = 0.0209 h^{-1}, doubling time = 33.16 h).

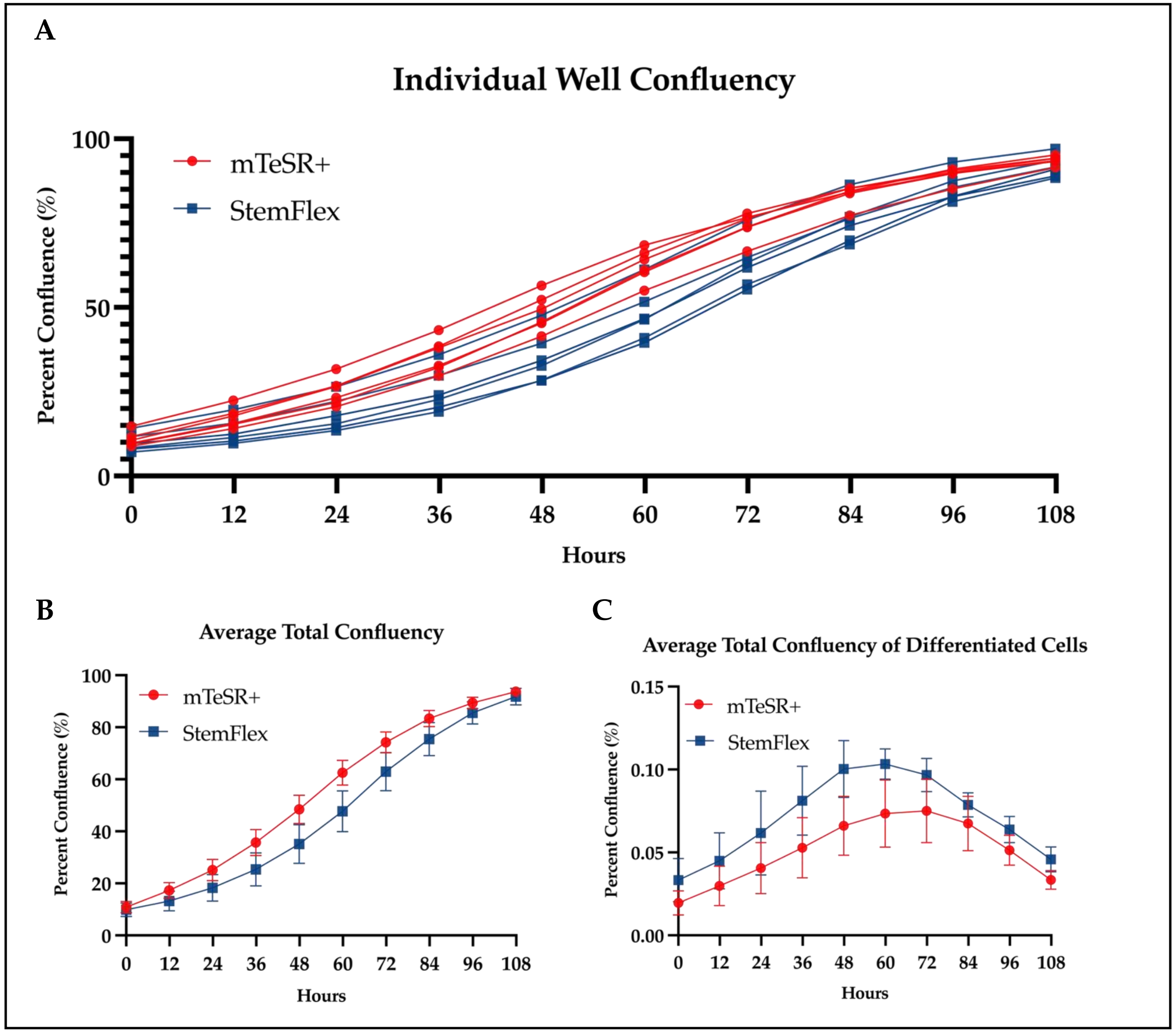

Supplementary Figure S2. Secondary quantitative analysis of hiPSC growth kinetics and differentiated cell burden in mTeSR+ and StemFlex media measured by automated live-cell imaging. iP11N cells were maintained in mTeSR+ (red, circles) or StemFlex (blue, squares) medium and imaged every 12 hours over 108 hours using the CellXpress.ai automated imaging platform at 4× magnification. Machine learning–based segmentation models were applied in IN Carta to quantify cellular growth and differentiation-associated metrics. **(A)** Individual well confluence trajectories for biological replicates cultured in mTeSR+ and StemFlex conditions. **(B)** Total confluence (%) over time. **(C)** Mean total confluence (%) of differentiated cells over time. Data are presented as mean ± SD.

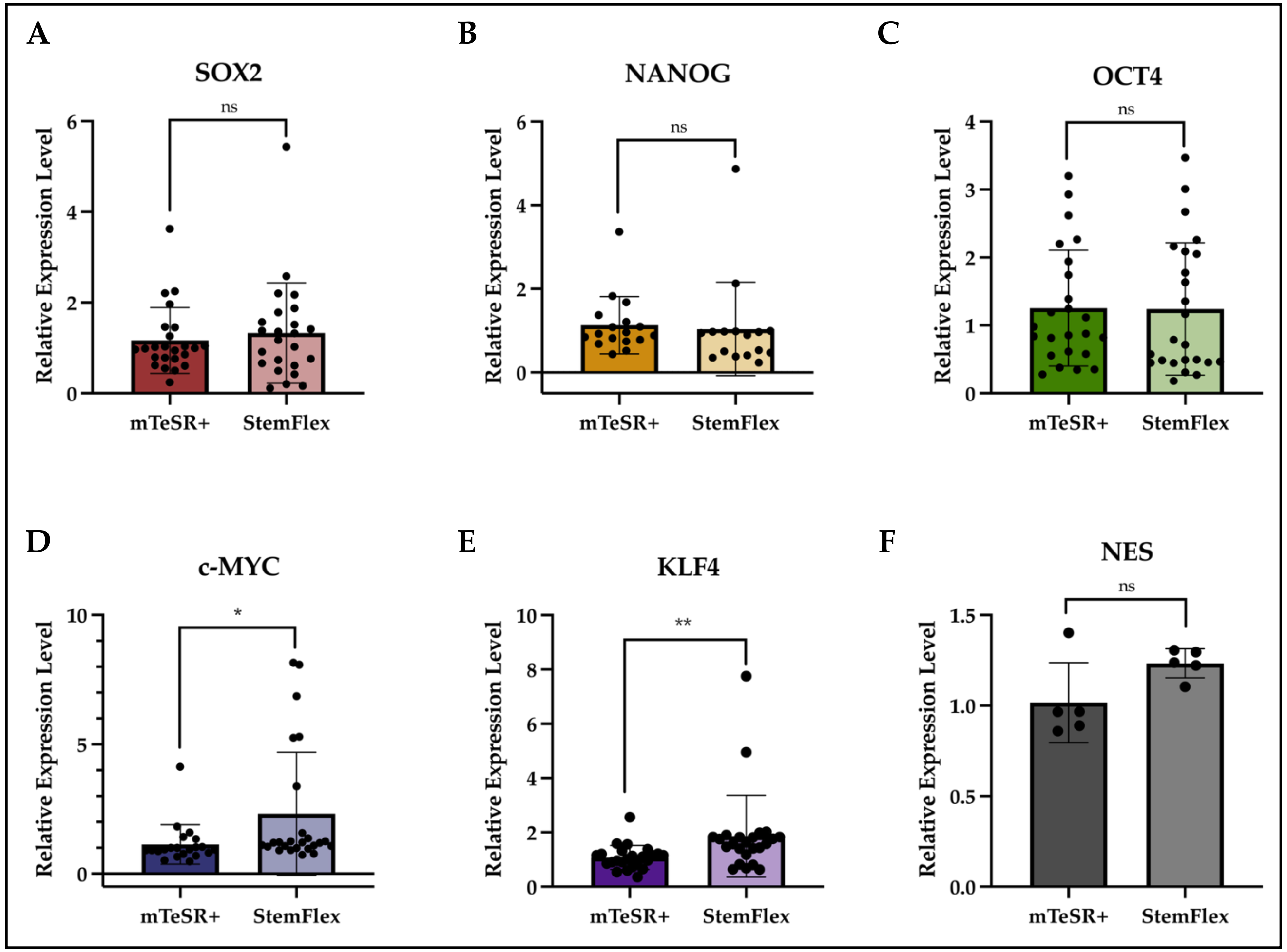

Supplementary Figure S3. Quantitative RT-qPCR analysis of pluripotency and neural marker gene expression in iP11N hiPSCs cultured in StemFlex or mTeSR+ medium prior to automated culture. Relative gene expression levels were normalized to a standardized amount of cDNA input. Data are presented as mean ± SD with individual data points shown. As several genes did not meet the assumption of a Gaussian distribution, statistical comparisons were performed using the Mann-Whitney U test, with resulting p-values corrected for multiple comparisons across the six genes assessed using the Holm-Bonferroni method; ns = not significant. **(A)** *SOX2* expression (mTeSR+ n=24, StemFlex n=24; adjusted p=1.000, ns). **(B)** *NANOG* expression (mTeSR+ n=17, StemFlex n=16; adjusted p=0.781, ns). **(C)** *OCT4* expression (mTeSR+ n=24, StemFlex n=24; adjusted p=1.000, ns). **(D)** *c-MYC* expression (mTeSR+ n=21, StemFlex n=25; adjusted p=0.032, *). **(E)** *KLF4* expression (mTeSR+ n=24, StemFlex n=24; adjusted p=0.007, **). **(F)** *NES* expression (mTeSR+ n=5, StemFlex n=5; adjusted p=0.603, ns). Statistically significant differences in expression were detected for c-MYC and KLF4, with all other genes remaining non-significant between culture conditions after correction for multiple comparisons.

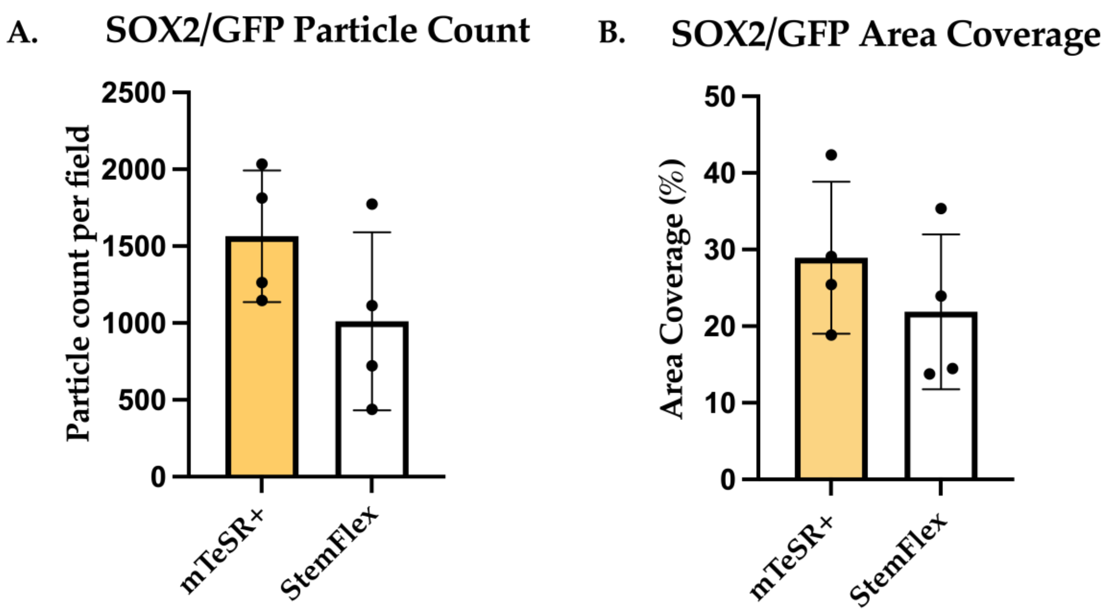

Supplementary Figure S4. Quantitative ImageJ analysis of SOX2-positive particle count and area coverage in iP11N hiPSCs cultured in mTeSR+and StemFlex media. Representative 20× ICC images from four wells per culture condition were analyzed using ImageJ. The SOX2/GFP channel was segmented using Otsu automatic thresholding followed by particle analysis. **(A)** SOX2-positive particle count per field and **(B)** percentage area (%Area) were quantified for mTeSR+ and StemFlex cultures. Data are presented as individual well values with mean ± SD (n = 4 wells per condition). StemFlex cultures showed a mean particle count of 1,012 ± 579 per field and mean area coverage of 21.88 ± 10.11%, compared with 1,565 ± 428 particles per field and 28.94 ± 9.90% area coverage for mTeSR+ cultures.
